# Screening of functional genes affecting the quality of translucent eggshell membranes based on RNA-seq analysis and DIA proteomics

**DOI:** 10.3389/fvets.2025.1583291

**Published:** 2025-05-19

**Authors:** Chun-Hao Han, Xiao-Yu Zhao, Chuan-Wen Wang, Li-Jun Xu, Hua-Ge Liu, Hui Chen, Rong-Yan Zhou, De-He Wang

**Affiliations:** ^1^College of Animal Science and Technology, Hebei Agricultural University, Baoding, China; ^2^Baoding Xingrui Agriculture and Animal Husbandry Development Co., Ltd., Baoding, China; ^3^College of Veterinary Medicine, Hebei Agricultural University, Baoding, China; ^4^Baoding Municipal Livestock Workstation, Baoding, China; ^5^Hebei Animal Husbandry and Veterinary Research Institute, Baoding, China

**Keywords:** chicken, translucent, eggshell membrane, transcriptome, DIA

## Abstract

The quality of eggshells holds substantial economic significance and serves as a critical selection criterion in poultry breeding. Eggshell translucency significantly impairs their aesthetic quality, which is structurally attributed to the thinning of the eggshell membrane or reduced tensile strength. In this study, 836 dwarf white hens were selected, with 45 hens each assigned to the opaque group and the translucent group. Grading for eggshell translucency was conducted at 75, 80, and 85 weeks of age. Based on the results from these three gradings, 35 hens that consistently produced translucent eggs and 35 hens that consistently produced opaque eggs were reclassified into the translucent group and the opaque group, respectively. The thickness of the eggshell membrane, latitudinal and longitudinal tensile force and length, and other indicators related to eggshell membrane quality were measured. Correlation analysis was performed using RNA-seq genomics and DIA proteomics based on the relationships among these indicators. Transcriptome analysis revealed 179 significantly differentially expressed genes, indicating that the causes of translucent eggshells are associated with metabolism, signal transduction, the immune system, molecular binding, transport, and catabolism. Seven potential candidate genes, including *CACNA2D2* and *KIF26A*, were identified. Proteomics analysis identified 565 differentially expressed proteins, suggesting that the expression of eggshell membrane proteins in translucent eggs is linked to protein metabolism, amino acid synthesis, immune regulation, metabolism and oxidative stress, catalytic activity, and molecular transport. Candidate proteins such as *SLC4A1*, *APOA1*, and *APOA4* were also identified. Combined proteomics and transcriptomics analysis identified 42 differentially expressed proteins and genes, including candidate genes such as *CA2*, *LUZP2*, *OVA*, *OVALY*, *OVST*, and *AVBD11*. This study provides a foundation for elucidating the genetic regulatory mechanisms underlying eggshell membrane quality and offers valuable references and directions for further enhancing eggshell quality.

## Introduction

Eggshell translucency manifests as watermark-like blemishes on the surface of the shell. This condition is likely to compromise the aesthetic integrity and commercial value of the eggs, thereby posing significant challenges for both marketability and consumer acceptance. Moreover, translucent areas increase the susceptibility of eggs to *Salmonella* contamination (1), posing serious food safety risks. A complete egg comprises the contents (egg yolk and egg white), the eggshell membranes (boundary membrane, inner eggshell membrane, and outer eggshell membrane), and the mineralized layers of the eggshell (mammillary layer, pore structures, vertical crystalline layer, and cuticle). Both the eggshell membranes and the mineralized layers play crucial roles in the formation of translucent eggshell (2). Eggshell membranes (ESM) contain 90% protein, 3% lipids, 2% sugars, and small amounts of minerals such as calcium and magnesium (3). Among the 90% of existing proteins, 472 kinds of protein types have been identified (4). These include some typical proteins with special structures, such as lysozyme, ovotransferrin, ovalbumin, and ovocledidin-17. The other part of the proteins are common ones like osteopontin and keratin (5). Studies on eggshell translucency have revealed significant differences in the mechanical properties (6), physical structure (7), and amino acid differences (8) of the eggshell membranes between translucent eggs and opaque eggs. However, the underlying mechanisms affecting eggshell membrane quality remain to be fully elucidated. The development of eggshell translucency is influenced by multiple factors, including environmental conditions, nutritional status, and genetic factors. It is likely that the formation of eggshell translucency results from the interaction of multiple differential traits or genes controlling these traits. Nonetheless, a clear understanding of the specific mechanisms involved has yet to be established. Therefore, this study aims to use RNA-seq technology and DIA proteomics technology to reveal the structural and regulatory basis of eggshell membrane differences, thereby providing certain molecular genetic-level reference basis for selecting and breeding normal eggshell membrane-type hens.

## Materials and methods

### Experimental hens

From a population of 836 dwarf white chickens subjected to seven generations of selection, 90 individuals (45 from the opaque group and 45 from the translucent group) were selected at 67 weeks of age, based on prior research findings (9). A four-level scoring system (10) was conducted on these chickens at 75, 80, and 85 weeks of age. Eggs from each chicken in the dwarf white group were collected over five consecutive days and labeled according to their cage numbers. Collection ceased once three eggs were obtained per chicken; if fewer than three eggs were collected within the five-day period, collection also stopped. After removing abnormal eggs, the remaining eggs were stored for 5 days before undergoing eggshell translucency grading. Based on the results of the three grading sessions, 70 dwarf white chickens were reclassified into two groups of 35 each: the opaque group (with opaque eggshell membrane quality) and the translucent group (with abnormal eggshell membrane quality).

### Eggshell membrane quality determination

The grading method used grayscale identification (11) to obtain translucent indicators, including total egg shell area (SUSHA), total translucent area (SUSA), translucent number (QS), average translucent area (AAES), and average translucent diameter (DS). The translucent rate (RSS) was calculated as the ratio of total translucent area to total eggshell area.

Egg weight, eggshell weight, eggshell strength, yolk weight, yolk color, Haugh Unit, eggshell thickness, and eggshell membrane thickness were measured on 15 eggs from each of the 15 test eggs of the selected opaque and translucent flocks. Egg weight, yolk color, and Haugh units were measured using an egg quality tester (EA-01, ORKA Ltd., Israel); eggshell thickness and eggshell membrane thickness were measured using a spiral micrometer (Kastemer Technology Development Co., Ltd., Beijing), and eggshell strength was measured using an eggshell strength tester (ESTG-01, ORKA, Israel). Egg Shape Index using Egg Shape Index Analyzer (NFN385, ORKA Technology Ltd., Hof Ashkelon 79165, Israel), egg shape index = short axis length (width) of egg/long axis length (height) of egg × 100%. The long axis is the maximum linear distance between the tip of the egg and the blunt end, and the short axis is the maximum transverse diameter in the middle of the egg (perpendicular to the direction of the long axis). The measurements were repeated 3 times for each egg and averaged.

The eggshell membrane toughness includes the eggshell membrane stretching force and stretching ratio. A small hole was drilled in the blunt end of the eggshell for the above measurement of egg quality, the egg white and yolk contents were poured out, the egg white residue on the inner membrane of the eggshell was washed with distilled water, and the eggshell and eggshell membrane were mechanically separated from the air chamber. For eggshell membrane toughness measurement, two 0.5 cm × 0.5 cm samples of the above opaque and translucent eggs were selected along the radial and longitudinal directions, and the selected eggshell membranes were washed and dried, and then the maximum tensile force and tensile ratio were measured with a dynamometer as the eggshell membrane toughness.

### Transcriptome and proteome of isthmus tissue

The egg-laying patterns of 70 dwarf white chickens, stratified into translucent and opaque groups based on eggshell characteristics, were systematically monitored and recorded at hourly intervals from 05:00 to 19:00. This comprehensive observation was conducted over a continuous 15-day period to ensure robust data collection. On the 16th day, 4 h post-oviposition (during the eggshell calcification stage), the white isthmus segment of the oviduct was collected. Based on the position of the eggs in the fallopian tubes and the thickness of the eggshell membranes, combined with the measurement data of the eggshell membrane quality, five hens with normal eggshell membranes and five hens with thinner eggshell membranes were selected from the opaque group and the transparent group, respectively, for subsequent transcriptome and proteome analyses. All experimental protocols involving terminal procedures shall be performed under direct supervision of a board-certified veterinary anesthesiologist, utilizing standardized pentobarbital sodium (USP-grade) anesthesia protocols followed by humane euthanasia through exsanguination during the pharmacologically determined unconsciousness phase (180–300 s post-induction).

### Isthmus tissue transcriptome

According to the manufacturer’s instructions, total RNA was extracted and purified using Trizol reagent (Life Technologies, Carlsbad, California, United States). mRNA was enriched using mRNA Capture Beads, followed by fragmentation at high temperature. The fragmented mRNA served as a template for first-strand cDNA synthesis in a reverse transcription system. Second-strand cDNA synthesis was performed simultaneously with end repair and A-tailing. Subsequently, adapters were ligated, and target fragments were selected and purified using Hieff NGS^®^ DNA Selection Beads. Finally, PCR amplification was conducted to construct the library, which was sequenced on the Illumina Novaseq X Plus platform.

After library construction, fastp (12) was used for quality control to filter out low-quality data, generating clean reads. These reads were then aligned to the ribosomal RNA (rRNA) database of *Gallus gallus* (chicken) using Bowtie2 (13) with zero mismatches allowed, and any reads mapped to rRNA were removed. The remaining unmapped reads were retained for subsequent transcriptomic analysis. Next, HISAT2 was employed to align the clean reads to the reference genome (*Gallus gallus*). Based on the HISAT2 alignment results, StringTie was used to reconstruct transcripts, and RSEM was applied to quantify gene expression levels in each sample. The expression levels are presented by the original reads count and FPKM. The original reads count represents the number of reads contained in the transcript. FPKM is the value corrected by sequencing depth and gene length. After obtaining the expression levels of all genes, we performed Principal Component Analysis (PCA) using R.^1^ The gene expression data were analyzed using the DESeq2 software (14), which consisted of three main steps: (I) normalization of read counts; (II) calculation of *p*-values based on statistical modeling and hypothesis testing; (III) multiple testing correction to obtain the false discovery rate (FDR). Finally, based on the differential expression analysis results, genes with FDR <0.05 and |log₂FC| >1 were identified as significantly differentially expressed genes (DEGs).

### Isthmus tissue proteomics

The isthmus tissues of the translucent and opaque groups were selected for sample pretreatment, including protein extraction, denaturation, reductive alkylation, enzymatic digestion and peptide desalting. In this experiment, the tissue samples were pretreated with iST sample pretreatment kit (PreOmics, Germany). The samples were milled in liquid nitrogen, 50 μL of lysis solution was added and heated at 95°C for 10 min at 1,000 rpm. The samples were cooled to room temperature, incubated with trypsin digestion buffer at 37°C for 2 h at 500 rpm, and the enzymatic reaction was terminated by adding termination buffer. The peptides were desalted using the iST cartridge in the kit, eluted with 2 × 100 μL elution buffer, and the eluted peptides were vacuum dried and stored at −80°C. After sample processing DDA characterization was performed to build the spectral database, the library building parameters were fixed modification: carbamidomethyl (C), variable modification: methionine oxidation. The false positive rate (FDR) was set to 1% for both parent ion and peptide levels; DIA data acquisition was also performed with mass spectrometry parameters set as (I) MS: scan range (*m*/*z*): 350–1,500; resolution: 120,000; AGC target: 4e6; maximum injection time: 50 ms; (II) HCD-MS/MS: resolution: 30,000; AGC target: 1e6; collision energy: 32; energy increase: 5%. (III) Variable window acquisition, 60 windows were set, overlapping serial ports were set, each window overlapped by 1 *m*/*z*.

After library building for protein qualitative and quantitative analysis, protein qualitative analysis required to meet the identification criteria of precursor threshold 1.0% FDR, protein threshold 1.0% FDR at the peptide and protein levels, respectively. Protein quantitative analysis was firstly performed by opaqueizing the overall sample spectral peak intensity using the local opaqueization method in Pulsar (15) software, and then the average of the peak areas of the top three MS1 peptides with FDR less than 1.0% were screened for protein quantification.

### Transcriptome-proteome joint analysis

The integrated analysis of transcriptome and proteome involves combining gene transcription level (mRNA) and protein expression level data. By calculating the expression correlation between mRNA and corresponding proteins using the Pearson correlation coefficient, researchers identified common differentially expressed genes/proteins (DEGs/DEPs) in both RNA-seq and proteomics datasets through Venn diagram analysis. These shared candidate genes/proteins were subsequently subjected to functional annotation to reveal their biological roles and regulatory mechanism.

### Analysis methods

Data were statistically analyzed using SPSS 22.0 for independent samples *t*-test. The test data were expressed as “mean ± standard deviation,” and *p* < 0.05 indicated significant differences, and *p* < 0.01 indicated highly significant differences. RNA-seq data were analyzed to identify differentially expressed genes (DEGs) between the translucent and opaque groups, with significance criteria set at *p* ≤ 0.05 and |log2FC| >1.

FPKM is the value corrected by sequencing depth and gene length. The formula for calculating FPKM is:


$$\text{FPKM}=\frac{10^{6}C}{\mathrm{NL}/10^{3}}$$

where *C* represents the number of fragments mapped to gene *i*, *N* represents the total number of fragments mapped to the reference genome, *L* represents the length of gene *i* (in base pairs).

In this study, we conducted a transcriptome-protein association analysis to investigate genes expressed at both the transcriptional and protein levels in the translucent and opaque groups. Since mRNA and protein represent gene expression at the transcriptional and translational levels, respectively, this integrated approach focuses on genes expressed in both datasets simultaneously. Using Venn diagram analysis, we identified genes that are expressed at both levels. The formula for Pearson correlation coefficient (*r*):


$$r=\frac{\mathrm{COV}\;(X_{\text{mRNA}},Y_{\text{protein}})}{\sigma_{X}*\sigma_{Y}}$$

Mathematically, COV represents the covariance between variables, σ denotes their standard deviations, while *X* and *Y* correspond to the log₂-transformed normalized expression values of mRNA and protein, respectively.

## Results

### Egg quality

The physical and quality characteristics of eggs from the dwarf white translucent group and the opaque group are summarized and compared in Table 1. As shown in Table 1, the key indicators measured, including egg weight (EW), eggshell weight (ESW), yolk weight (YW), and eggshell strength (ESS), showed no statistically significant differences between the two groups.

**Table 1:** Comparison of egg quality between opaque and translucent groups in dwarf white hen.

Item	Opaque group	Translucent group	*p*
Translucent grade	1.98 ± 0.80	3.33 ± 0.74	0.00
Egg weight (g)	50.87 ± 13.75	51.31 ± 13.52	0.63
Eggshell weight (g)	6.14 ± 0.55	6.18 ± 0.52	0.14
Egg yolk weight (g)	14.39 ± 1.05	14.87 ± 1.11	0.23
Egg white weight (g)	29.99 ± 3.47	30.17 ± 3.55	0.15
Haugh unit	81.77 ± 3.88	82.04 ± 4.17	0.22
Egg shape index	1.31 ± 0.04	1.31 ± 0.04	0.61
Eggshell thickness (μm)	307.93 ± 27.88	305.22 ± 25.62	0.36
Eggshell membrane thickness (μm)	27.23 ± 4.11	23.10 ± 4.44	0.00
Eggshell strength (kg/cm^2^)	35.75 ± 8.50	31.89 ± 9.07	0.17
Egg yolk color	5.50 ± 0.82	5.40 ± 0.82	0.70

### Eggshell membrane quality

A detailed comparison of eggshell membrane toughness parameters between the translucent and opaque groups is presented in Table 2. The results demonstrated that both the latitudinal and longitudinal tensile forces of the eggshell membrane in the translucent group were significantly lower than those in the opaque group (*p* < 0.001). However, no significant differences were observed in either the latitudinal or longitudinal maximum stretch ratios between the two groups.

**Table 2:** Eggshell membrane toughness between opaque and translucent groups in dwarf white hen.

Item	Opaque group	Translucent group	*p*
Latitudinal direction maximum stretch ratio (%)	40.17 ± 13.21	39.28 ± 12.24	0.45
Latitudinal stretching force (N)	0.12 ± 0.01	0.11 ± 0.01	0.00
Longitudinal stretching maximum stretch ratio (%)	50.10 ± 15.37	49.95 ± 15	0.39
Longitudinal stretching force (N)	0.15 ± 0.02	0.13 ± 0.01	0.00

### Transcriptome analysis

Egg-laying times were recorded over 15 consecutive days for both the translucent and opaque groups of dwarf white hens, with detailed results presented in Table 3. At the time of sampling, eggs from both groups were in the white isthmus, and no significant differences were observed in eggshell membrane thickness.

**Table 3:** Statistical table of sample selection for the isthmus test.

Sample	Sampling location	Egg-laying interval (h)	Eggshell membrane thickness (μm)
Translucent-1	White isthmus	26.60	0.012
Translucent-2	White isthmus	26.80	0.011
Translucent-3	White isthmus	25.00	0.012
Translucent-4	White isthmus	25.60	0.013
Translucent-5	White isthmus	25.80	0.012
Opaque-1	White isthmus	24.50	0.011
Opaque-2	White isthmus	26.11	0.011
Opaque-3	White isthmus	25.25	0.012
Opaque-4	White isthmus	25.45	0.012
Opaque-5	White isthmus	26.00	0.011

Sequence quality metrics indicated high data integrity, with Q20 scores ranging from 97.13 to 97.62% and Q30 scores from 92.56 to 93.30%. Following quality control, an average of 48,937,515 clean reads per sample was obtained. Base quality distribution details are summarized in Table 4.

**Table 4:** Transcriptome information of opaque and translucent individuals.

Sample	Raw data (bp)	Clean data (bp)	GC (%)	Q20 (%)	Q30 (%)
Translucent-1	7,455,303,400	7,372,580,817	47.76%	97.62%	93.25%
Translucent-2	9,011,210,700	8,906,651,023	47.71%	97.21%	92.75%
Translucent-3	8,000,191,500	7,923,111,917	47.97%	97.22%	92.70%
Opaque-1	8,634,431,900	8,547,965,518	47.45%	97.32%	92.92%
Opaque-2	5,973,549,900	5,915,168,676	48.12%	97.17%	92.70%
Opaque-3	6,036,121,500	5,976,950,586	47.59%	97.13%	92.56%

**Figure 1:**
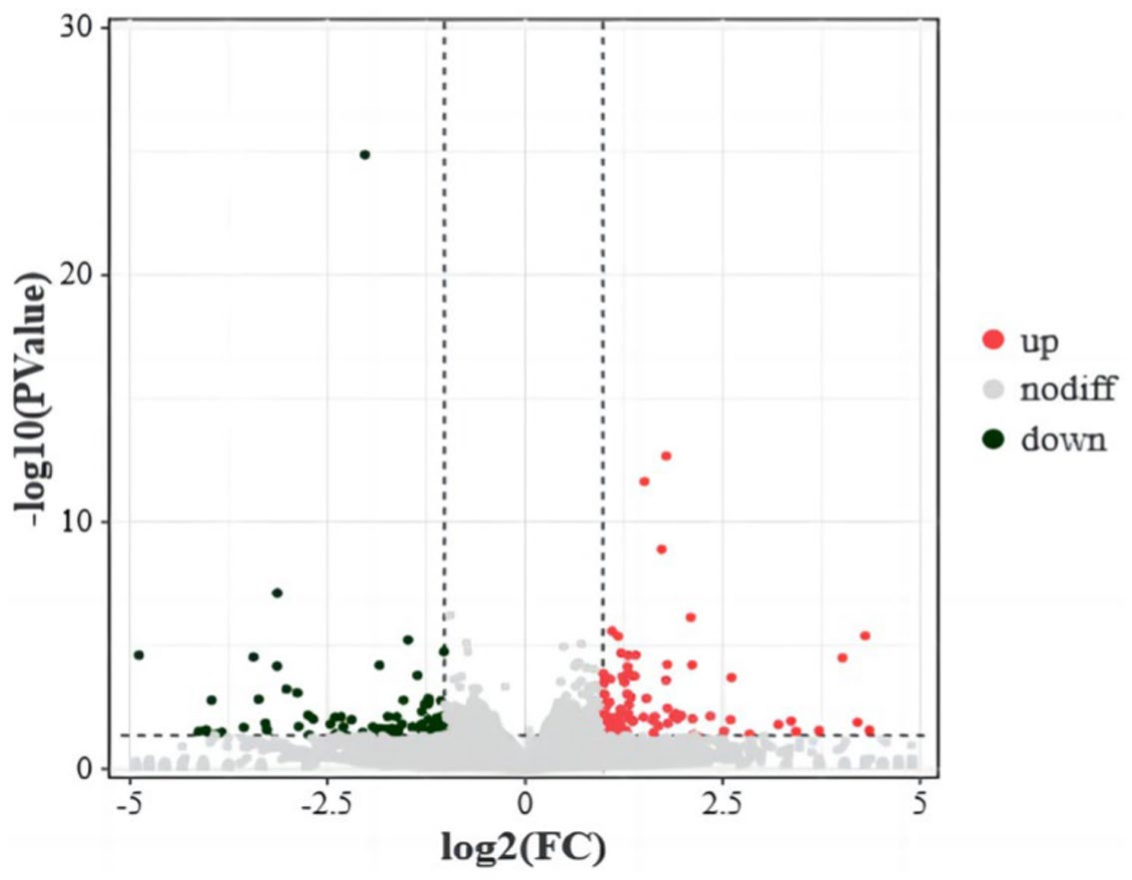
Differential gene volcanoes in the isthmus transcriptome of the translucent group and opaque group. The horizontal coordinates indicate the fold change of protein expression in different groups, the vertical coordinates indicate the negative log_10_ value of FDR or *p*-value for the difference between two subgroups, black on the left side represents down-regulated genes, red on the right side represents up-regulated genes, and gray represents genes with no significant difference.

RNA sequencing data analysis revealed a total of 16,666 genes, among which 179 were identified as DEGs. Specifically, 92 genes were significantly upregulated and 89 genes were significantly downregulated in the translucent group compared to the opaque group (Figure 1).

**Figure 2:**
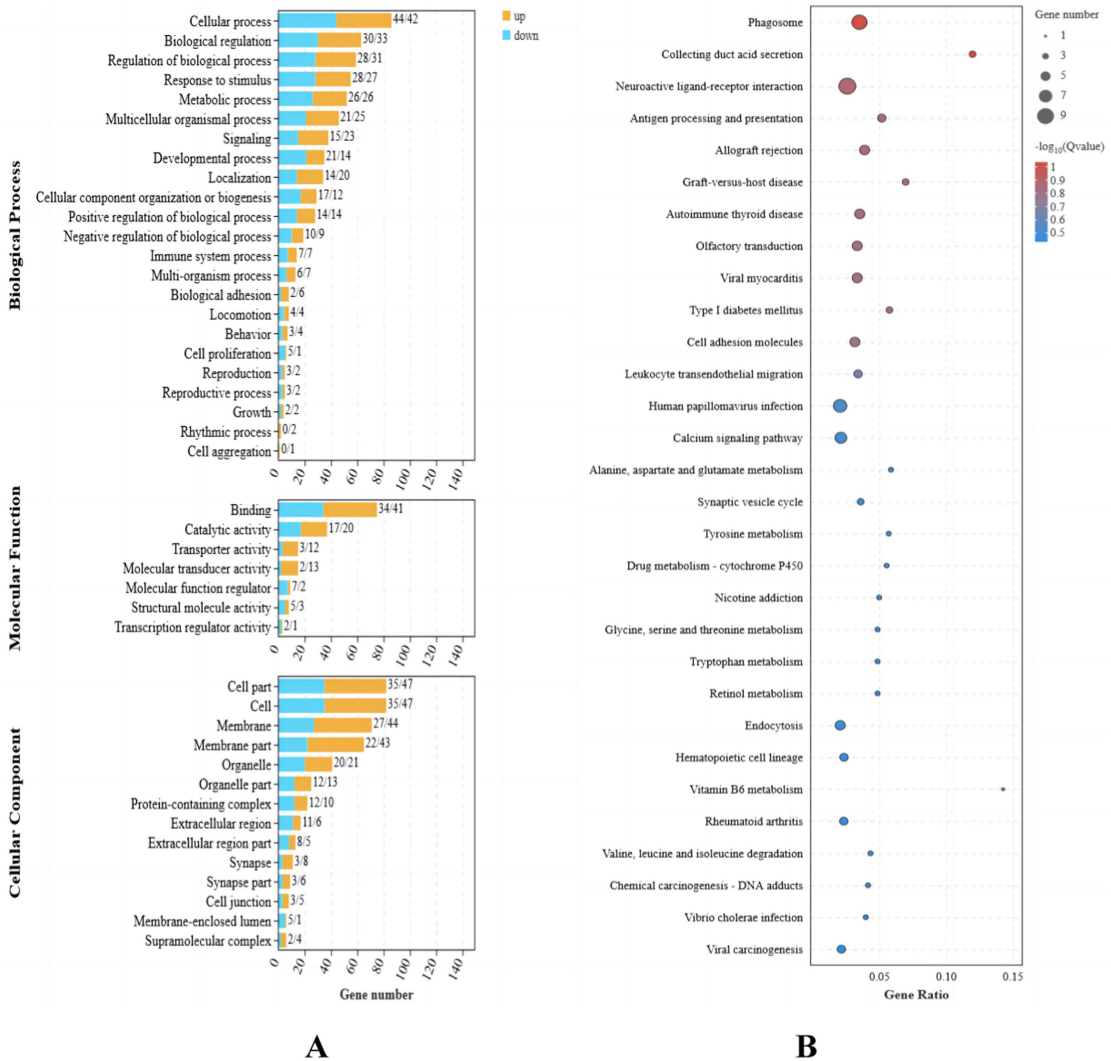
Transcriptome differential gene analysis of the translucent group and opaque group isthmus. **(A)** Transcriptome differential gene GO analysis. **(B)** Transcriptome differential gene KEGG enrichment analysis bubble map.

Functional enrichment analysis was performed on the nine differentially expressed genes using Gene Ontology (GO). A total of 37 GO terms were enriched, distributed across three functional groups: 21 for biological process (BP), 11 for cellular component (CC), and five for molecular function (MF). The most significantly enriched GO terms are illustrated in Figure 2A. Among these, the highest enrichment was observed for “cellular processes” (GO:0009987) in the BP category, “binding” (GO:0005488) in the MF category, and “cell” (GO:0005623) and “membrane” (GO:0016020) in the CC category.

Kyoto Encyclopedia of Genes and Genomes (KEGG) pathway enrichment analysis identified 13 significantly enriched pathways in the translucent and opaque groups, as shown in Figure 2B. The most abundant signaling pathways were prominently represented in this analysis.

**Figure 3:**
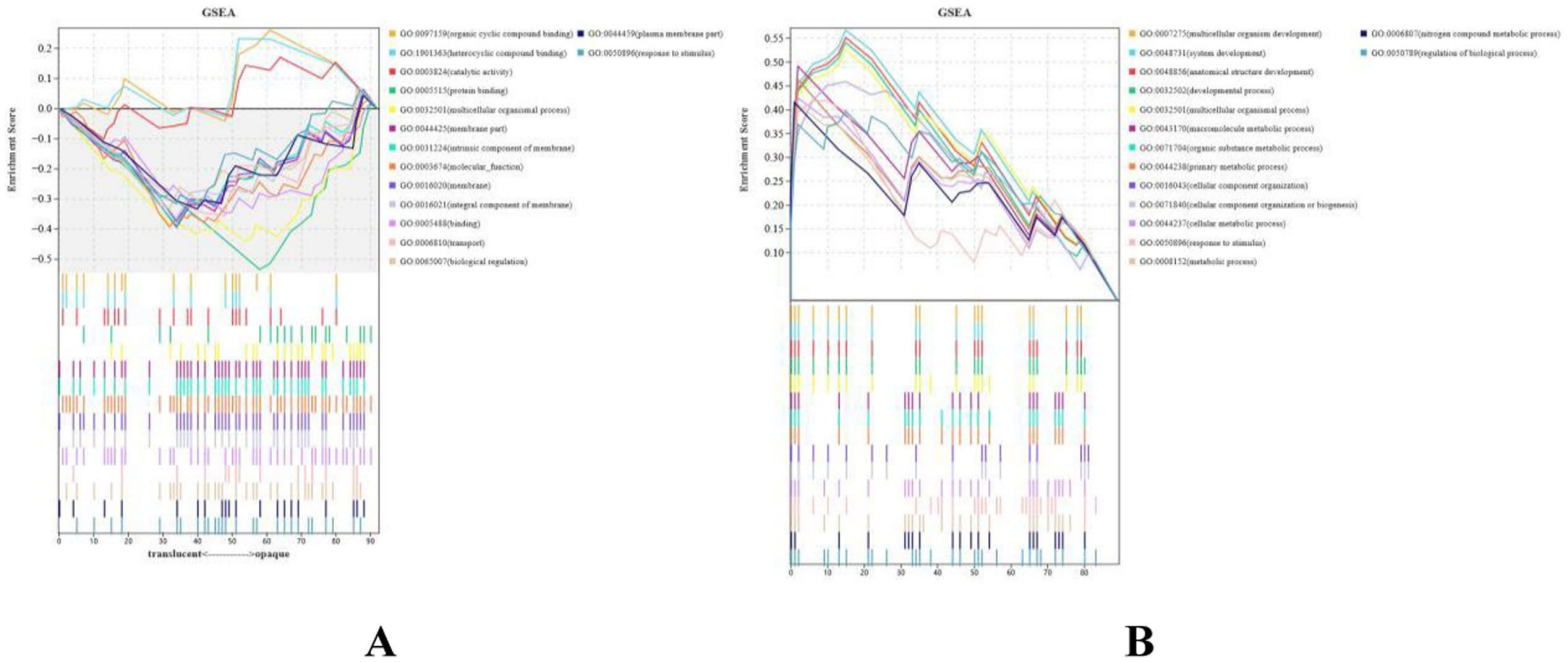
Protein identification statistics of translucent group and opaque group isthmus. **(A)** Running enrichment scores for upregulated gene sets in the translucent egg group. Colored lines represent different pathways. The middle panel shows gene positions within each set, and the bottom panel shows the ranked gene list. **(B)** Running enrichment scores for downregulated gene sets in the translucent egg group. Colored lines represent different pathways. The middle panel shows gene positions within each set, and the bottom panel shows the ranked gene list.

The 181 identified DEGs were used to perform Gene Ontology (GO) analysis for functional annotation, and gene set enrichment analysis (GSEA) was conducted to explore their function. The GSEA revealed significant upregulation of pathways related to organic cyclic compound binding, heterocyclic compound binding, and protein binding (Figure 3A), while pathways associated with multicellular organism development, system development, and developmental processes were notably downregulated (Figure 3B).

### Isthmus tissue DIA proteome analysis

**Figure 4:**
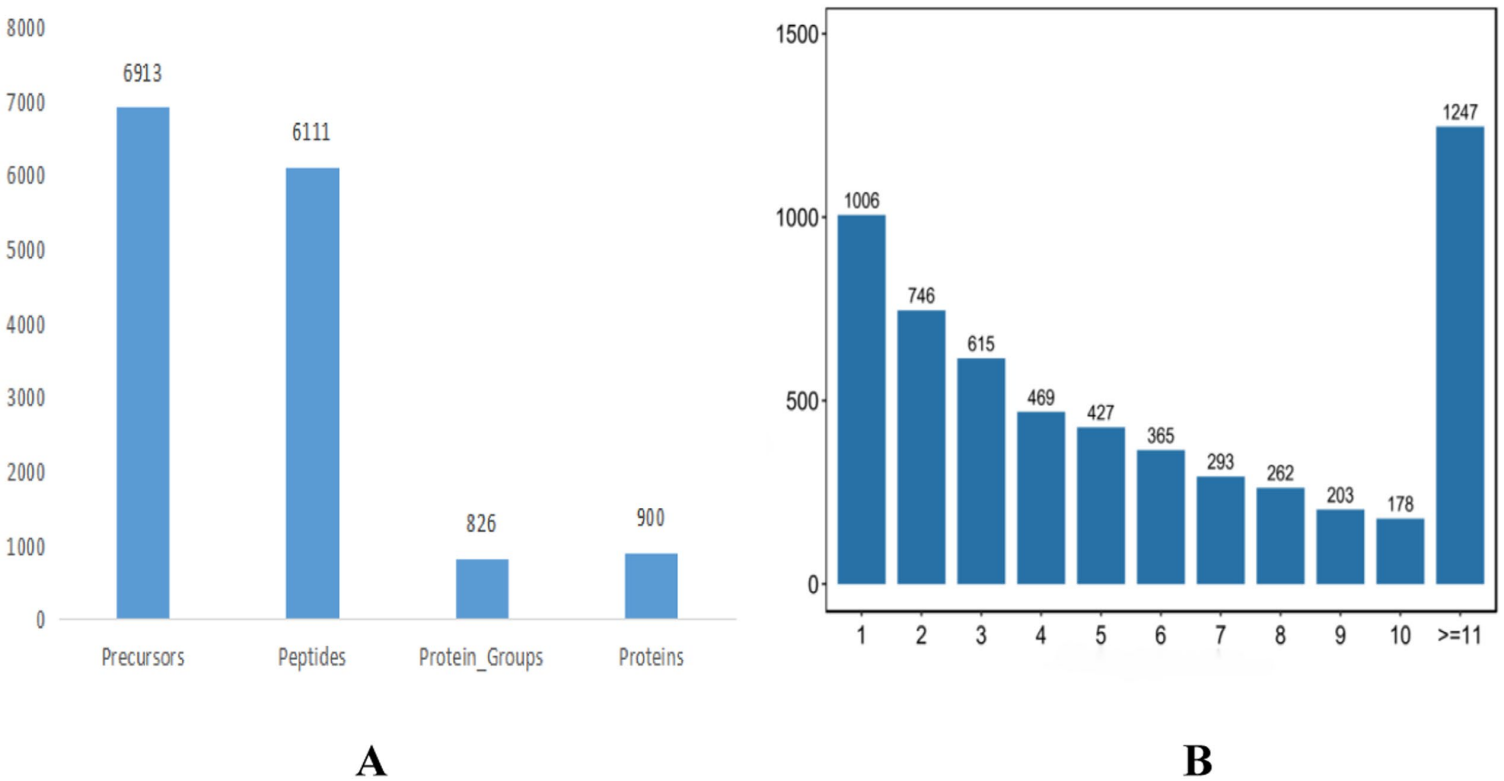
Protein identification statistics of translucent group and opaque group isthmus. **(A)** Protein identification statistics. **(B)** Peptide identification statistics.

In this study, the DIA (data-independent acquisition) technique was employed for both absolute and relative quantification of proteins. The proteomic analysis yielded a total of 46,973 matched spectra, leading to the identification of 42,827 peptides and 5,882 quantifiable proteins using the DIA proteome software tool (Figure 4A). The distribution of identified peptide bands is detailed in Figure 4B.

**Figure 5:**
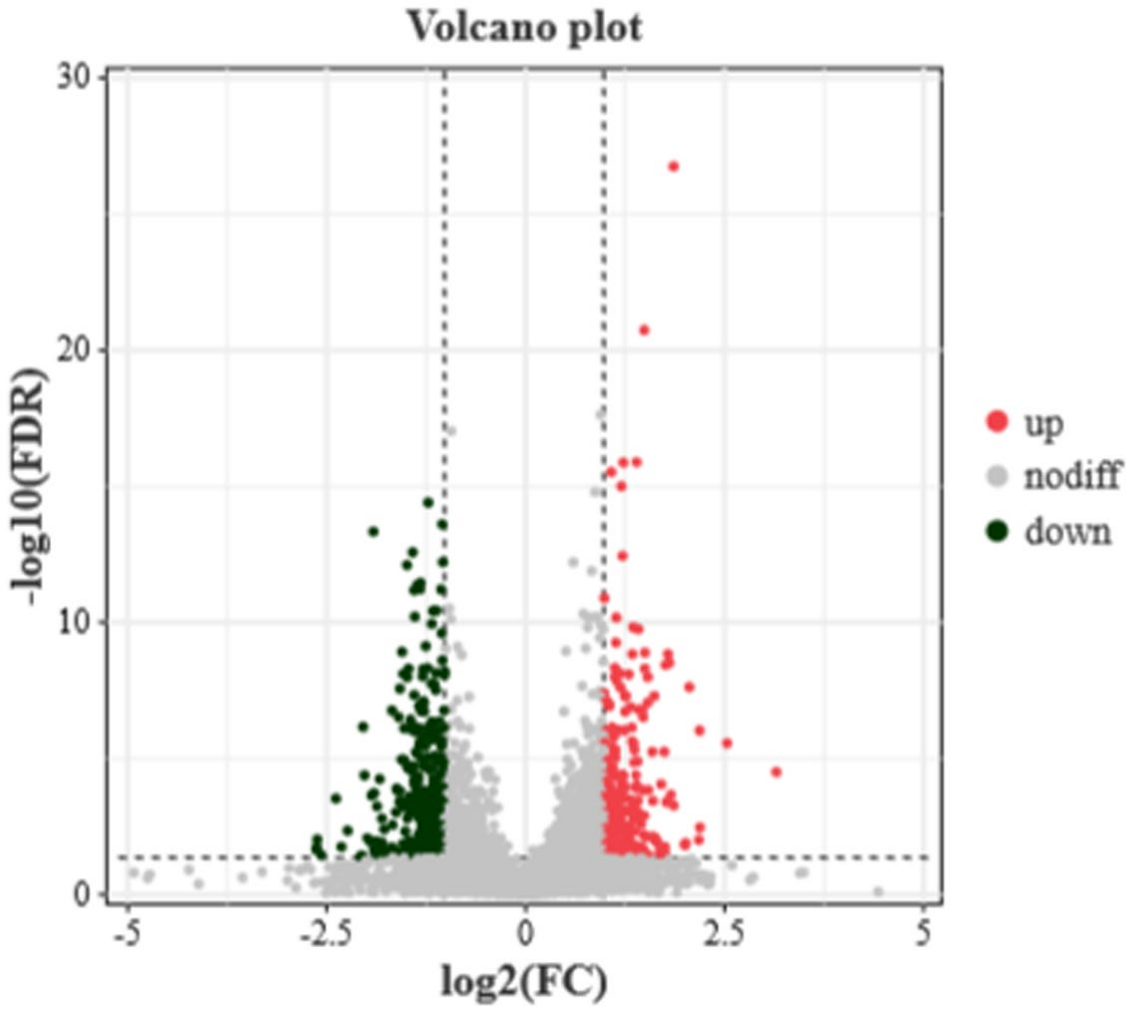
Differential protein volcanoes in the isthmus proteome of the translucent group and opaque group. Horizontal coordinates indicate the fold change in expression of proteins in different groups, vertical coordinates indicate the negative log_10_ value of the FDR or *p*-value of the difference between the two subgroups, black on the left side represents down-regulated proteins, red on the right side represents up-regulated proteins, and gray represents proteins with no significant difference.

To identify proteins with differential expression between the translucent and opaque groups, the dataset was further analyzed. Proteins were filtered using a fold change (FC) cutoff of >2 and a corrected *p*-value (*Q*-value) of <0.05. As detailed in Figure 5, a total of 565 differential proteins were identified in the isthmus tissues of the translucent and opaque groups, with 249 proteins upregulated and 316 proteins downregulated.

**Figure 6:**
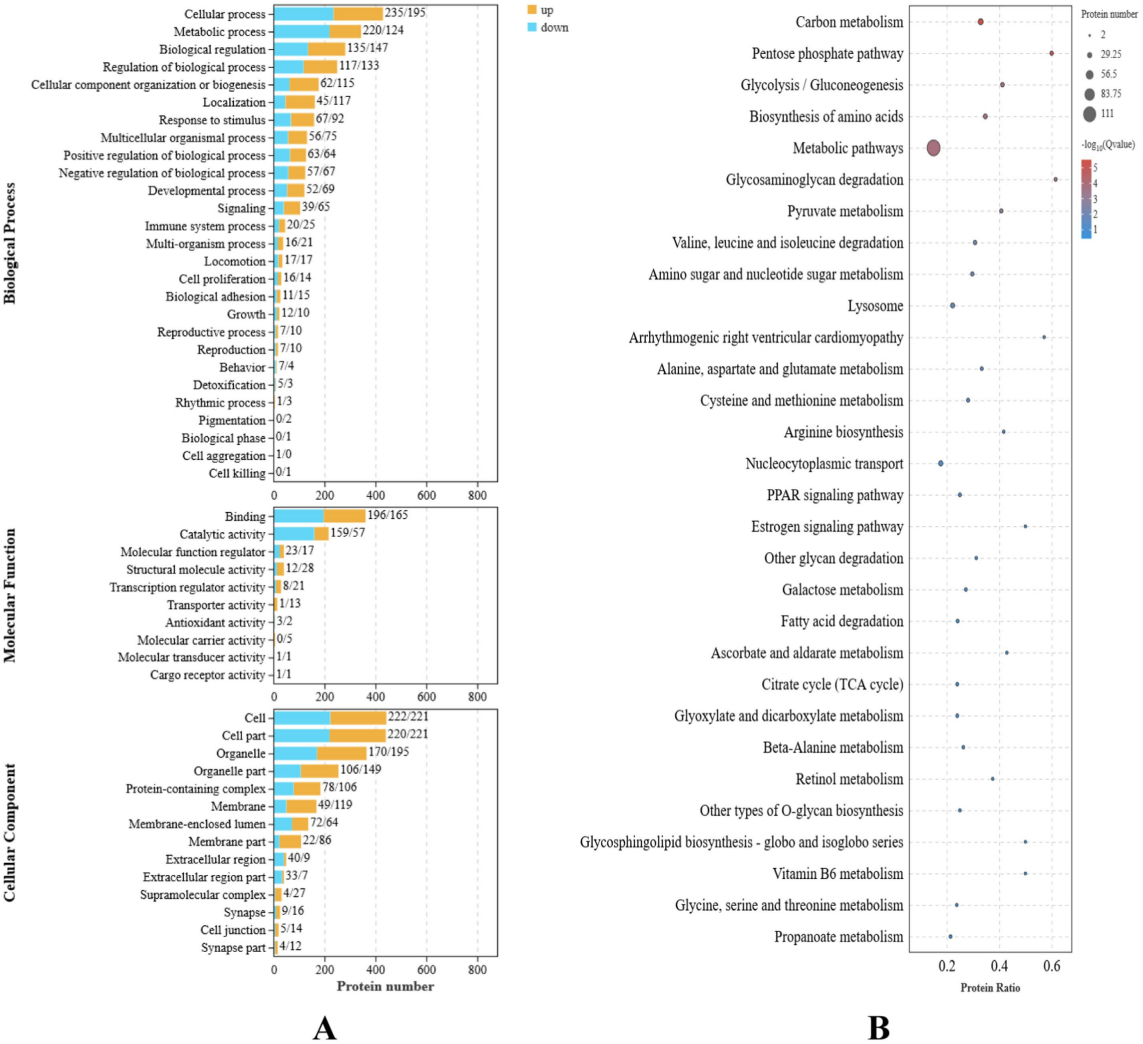
Plot of differential protein enrichment analysis of the isthmus proteome. **(A)** Secondary histogram of GO enrichment classification. **(B)** Bubble plot of significance of differential protein KEGG enrichment.

GO functional enrichment analysis of the differential proteins revealed significant enrichment in several biological processes, including carboxylic acid metabolism, organic acid metabolic processes, molecular redox regulation, chromosome assembly, DNA assembly, and protein assembly. The functional interaction network highlighted key gene enrichments such as transport activity, catalytic activity, antioxidant activity, and positive regulation of metabolic processes (Figure 6A).

To systematically investigate the mechanisms underlying the translucent phenotype, the differential proteins were further annotated using the KEGG (Kyoto Encyclopedia of Genes and Genomes) pathway database. The KEGG enrichment analysis, shown in Figure 6B, revealed that these proteins were primarily involved in processes such as protein processing in the endoplasmic reticulum, regulation of actin, nucleoplasm transport, positive regulation of carbohydrate synthesis, and amino acid biosynthesis.

**Figure 7:**
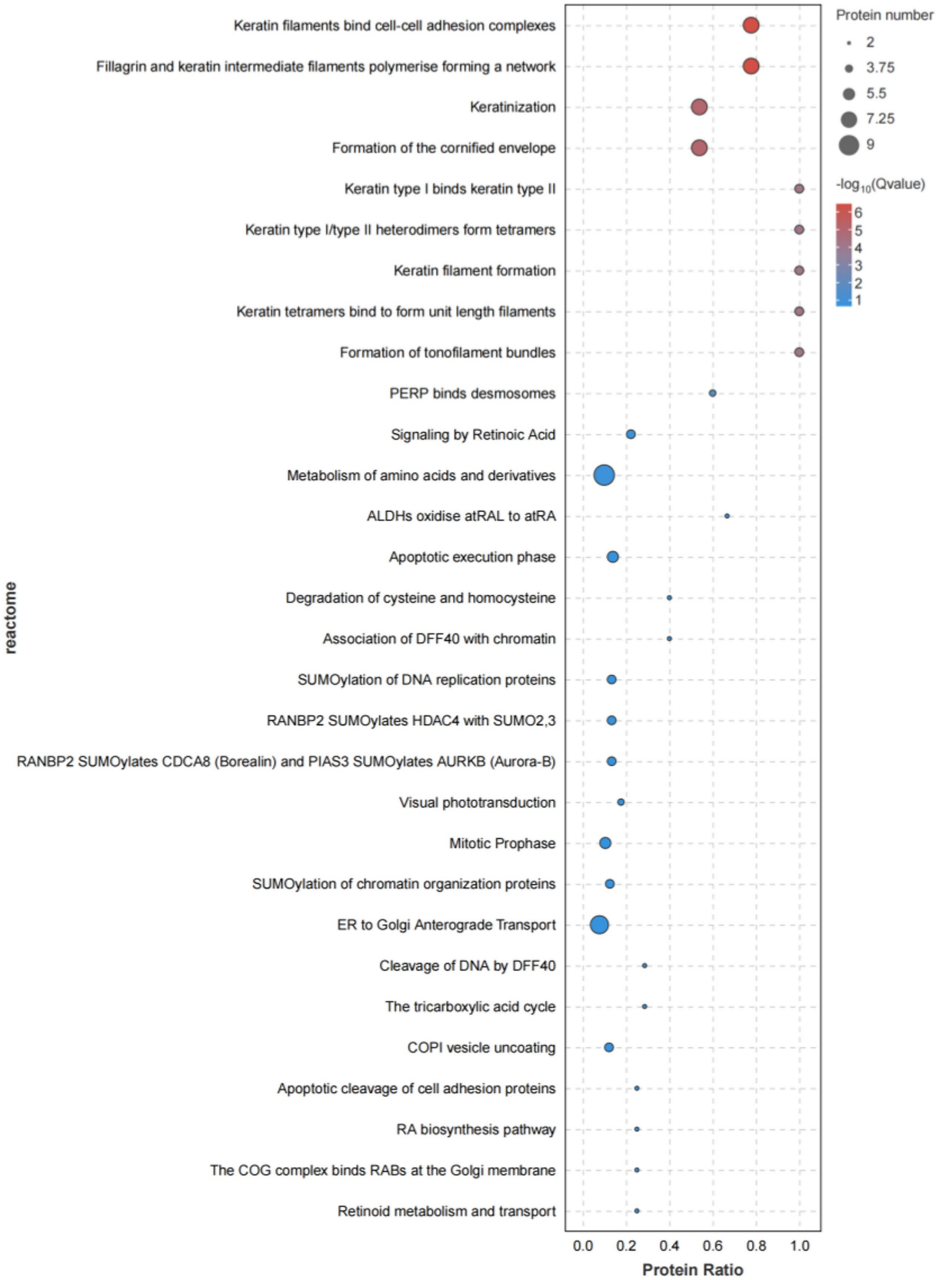
Bar chart of reactome enrichment for isthmus proteome differential proteins.

Reactome pathway enrichment analysis, as highlighted in Figure 7, demonstrated that these genes were predominantly enriched in pathways related to protein metabolism, immune regulation, protein modification, membrane transport, the mitotic cycle, and RNA metabolism.

### Joint analysis

**Figure 8:**
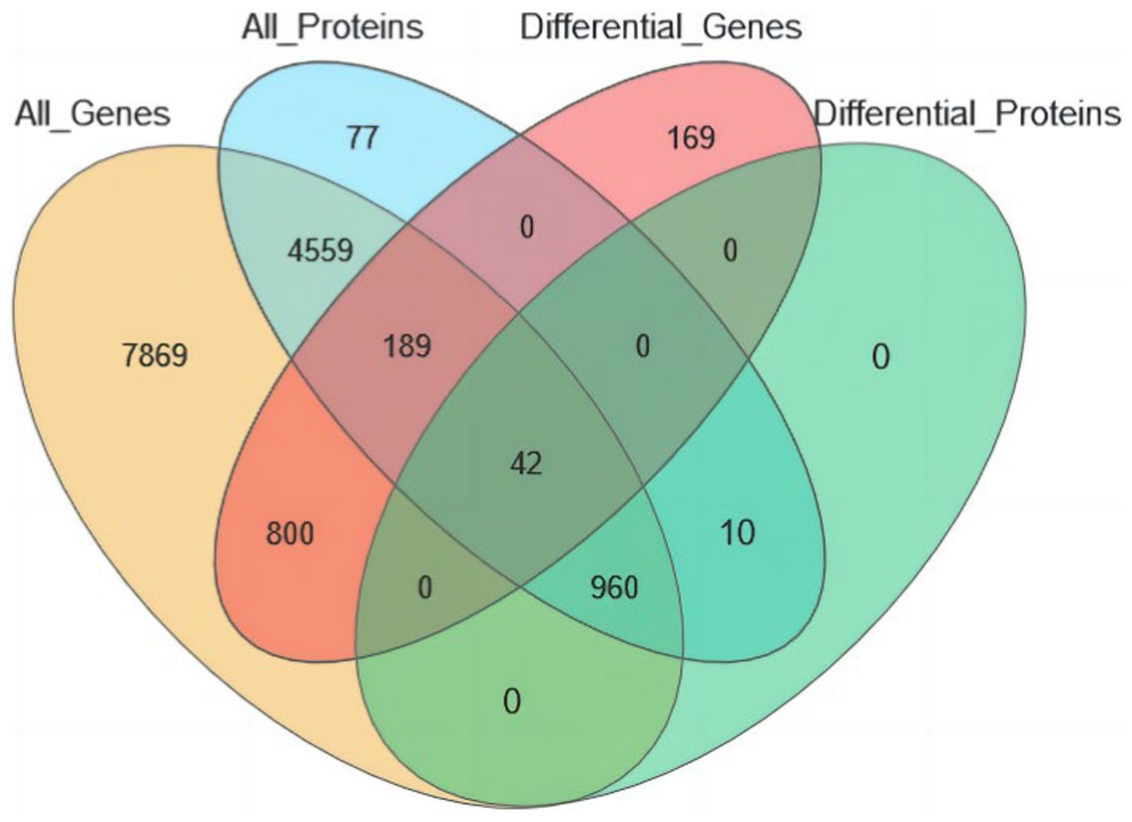
Venn diagram of transcriptome-proteome association analysis.

The joint analysis of the eggshell membrane proteome and isthmus tissue transcriptome revealed that 14,419 proteins with corresponding transcripts were identified in the transcriptomic data, while 5,837 proteins were detected in the proteomic data. To identify common differential genes and proteins, we applied the following criteria: genomic *p* ≤ 0.05 and a fold change of 2 for transcriptomic data, and proteomic FDR ≤0.05 and a fold change of 1.2 for proteomic data. This analysis yielded a total of 42 common differential genes and proteins (Figure 8).

**Figure 9:**
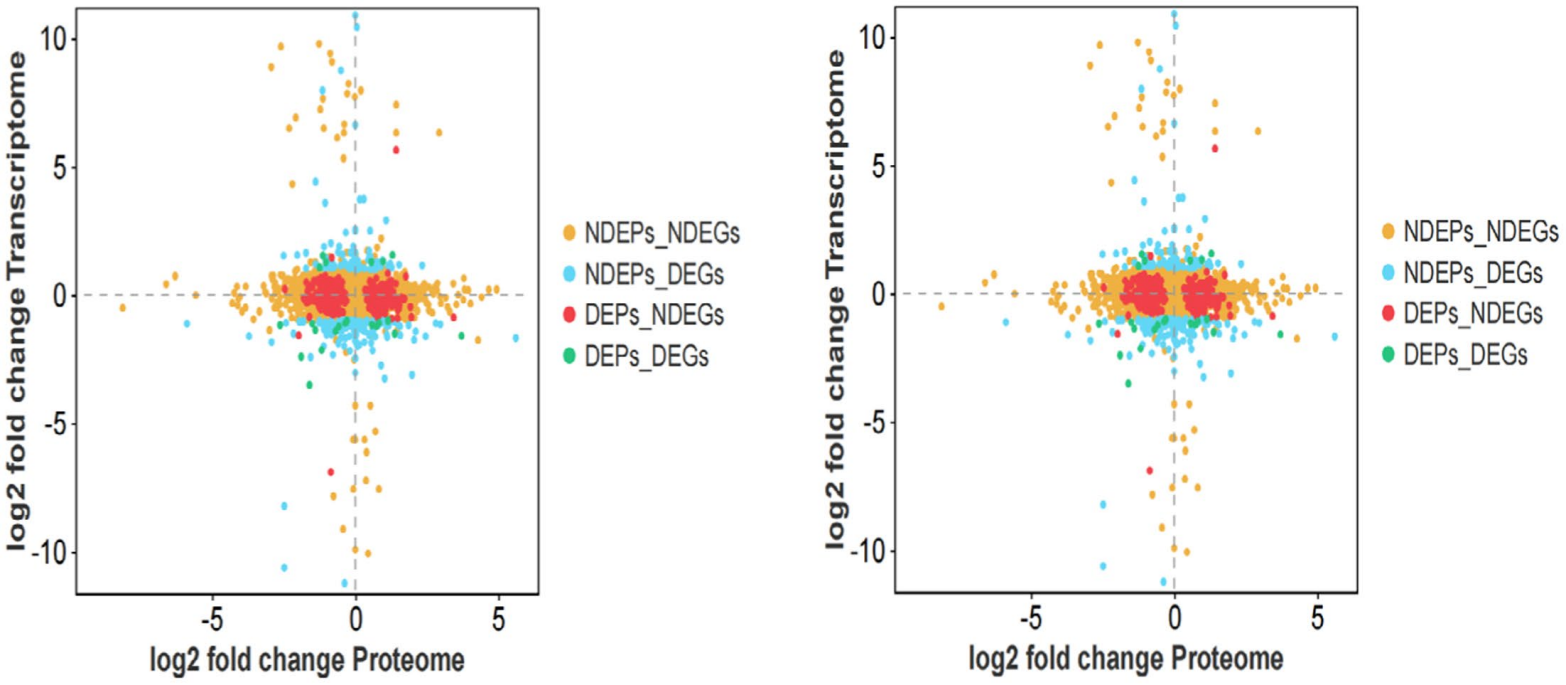
Four-quadrant and nine-quadrant maps of isthmus transcriptome-proteome association analysis.

To characterize the expression patterns of genes in both histologies, we generated four-quadrant and nine-quadrant maps. The four-quadrant map classifies genes expressed in both histologies into four categories based on their differential expression status: (1) DGEs_DEPs (genes significantly differentially expressed in both transcriptome and proteome), (2) NDGEs_DEPs (genes non-significantly differentially expressed in transcriptome but significantly differentially expressed in proteome), (3) DGEs_NDEPs (genes significantly differentially expressed in transcriptome but non-significantly differentially expressed in proteome), and (4) NDGEs_NDEPs (genes non-significantly differentially expressed in both transcriptome and proteome). The nine-quadrant map further categorizes genes into nine types according to their differential expression status in both histologies, providing a more detailed representation of the data (Figure 9). Linear regression analysis of the transcriptomic and proteomic profiles revealed a Pearson correlation coefficient of −0.0531.

**Figure 10:**
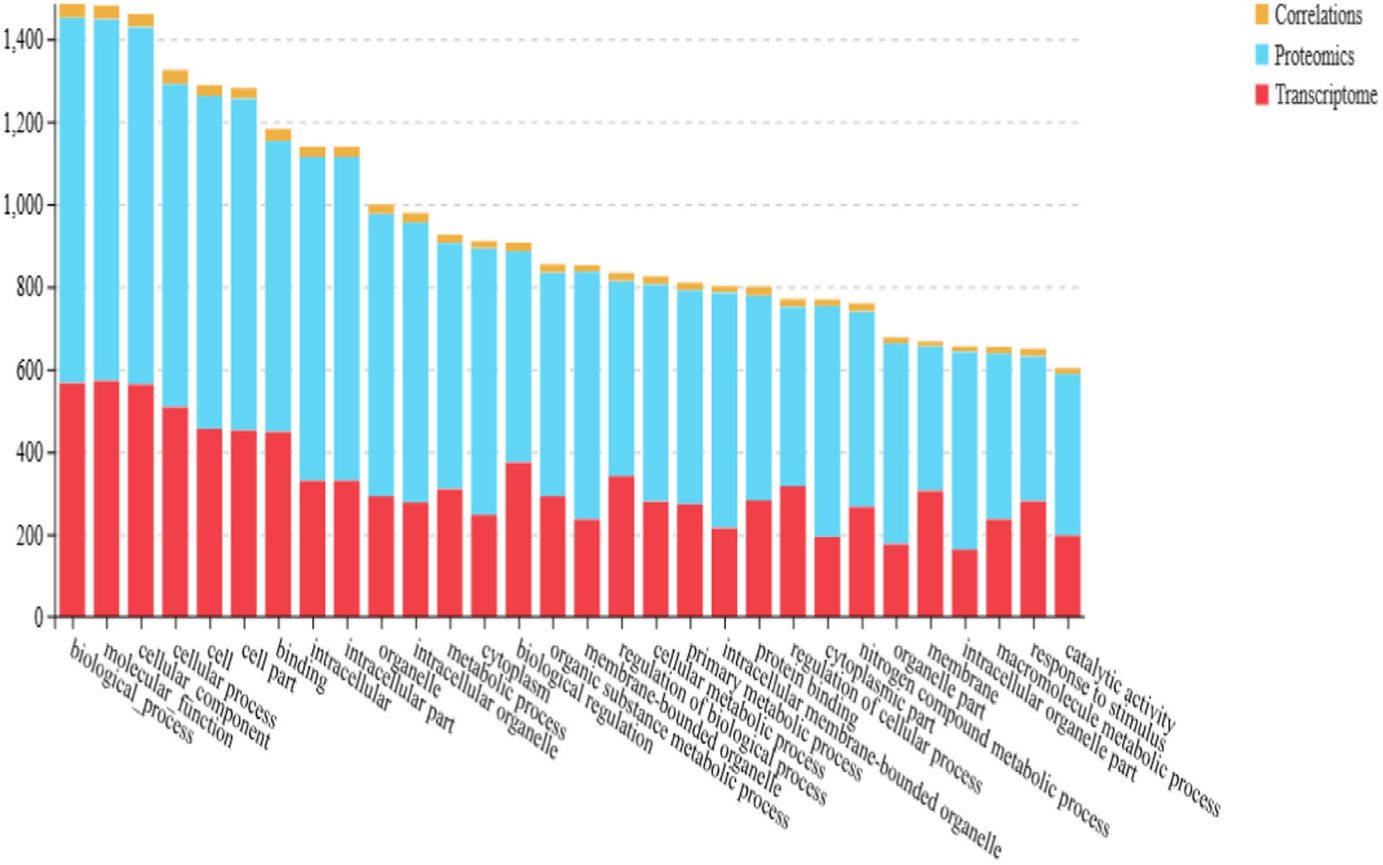
Isthmus transcriptome-proteome GO term association analysis graph.

The results of the joint GO functional enrichment analysis of the differential protein and gene sets are presented in Figure 10. The differential proteins and genes were primarily enriched in functional categories related to cellular processes, cellular components, molecular binding, and intracellular components.

**Figure 11:**
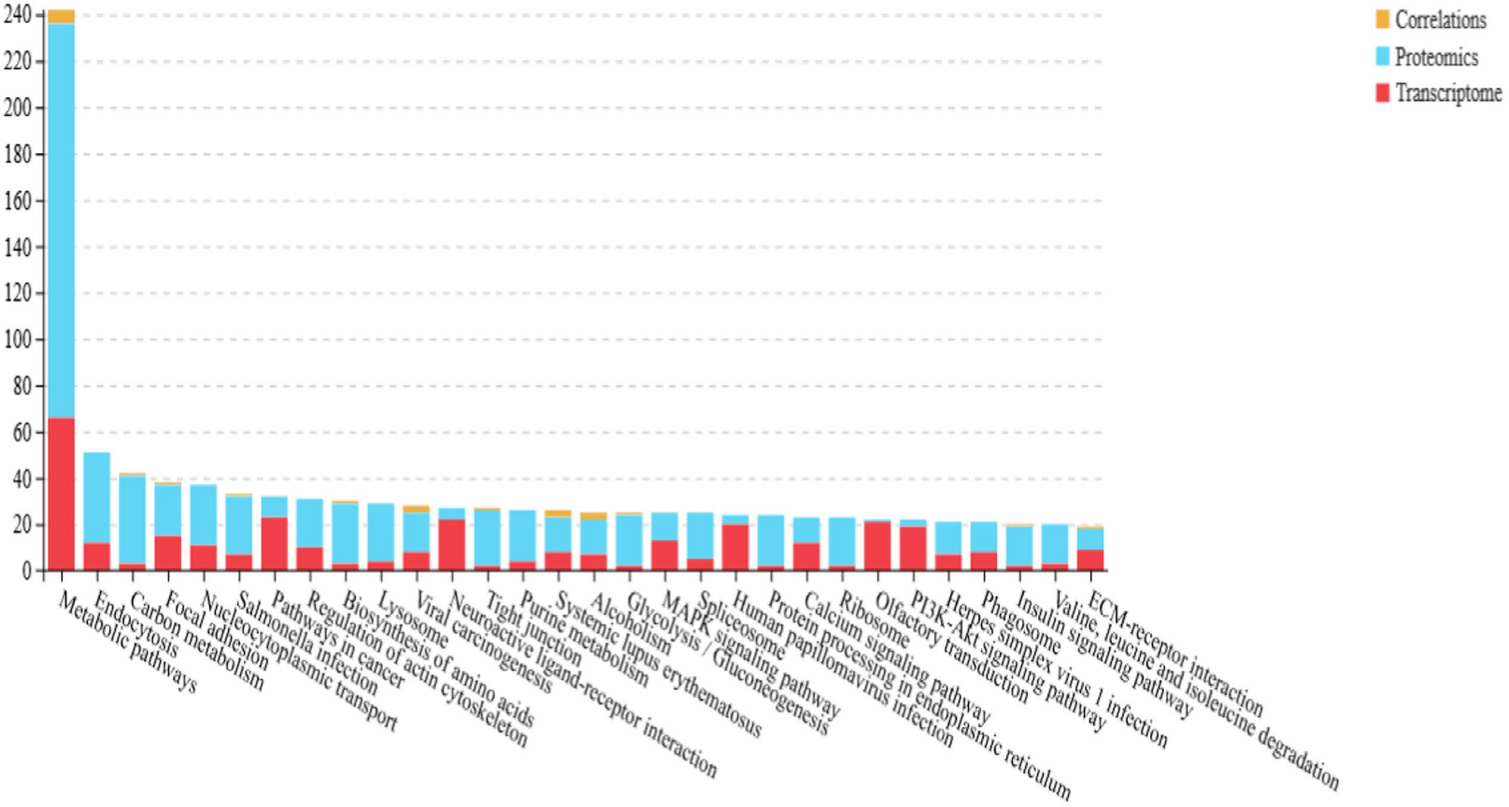
Isthmus transcriptome-proteome pathway association analysis.

The joint pathway enrichment analysis of the differential protein and gene sets is shown in Figure 11. The differential proteins and genes were mainly enriched in pathways associated with metabolic functions, endocytosis, and carbon metabolism.

## Discussion

### Eggshell membrane quality

Based on previous studies, variations in the chemical composition of the eggshell membrane can lead to changes in its physical structure, which ultimately influence the formation of eggshell translucency (16). In this experiment, the eggshell membrane thickness in the translucent group of dwarf white hens was significantly lower than that in the opaque group. Additionally, the opaque group exhibited significantly higher latitudinal and longitudinal tensile forces compared to the translucent group, while no significant differences were observed in the latitudinal and longitudinal tensile lengths. These findings align with previous research on eggshell translucency in dwarf brown-shelled hens (17). In this study, the opaque group exhibited significantly lower values for egg weight, eggshell weight, yolk weight, and albumen weight compared to the translucent group. Notably, egg weight—primarily influenced by hen body weight (18)—served as a key determinant of these differences, suggesting that the translucent trait may indirectly affect the physiological status of laying hens. Given that egg weight impacts eggshell weight, yolk weight, and albumen weight (15), the observed variations in these parameters likely stemmed from the disparity in egg weight between the two groups. Interestingly, the opaque group demonstrated superior eggshell strength relative to the translucent group. This phenomenon may be attributed to the structural integrity between the eggshell membrane and eggshell papillae (19), with potential contributions from variations in eggshell membrane thickness.

### Transcriptome

In this experiment, RNA sequencing (RNA-seq) was performed on oviductal isthmus tissues collected during eggshell membrane formation in both the translucent and opaque groups of dwarf white hens. A total of 16,666 genes were detected, including structural proteins such as collagen (e.g., *COL10A1*, *COL17A1*, and *COL3A1*), fibronectin (e.g., *FBN1* and *FBN2*), and lysyl oxidase homologs (*LOXL1* and *LOXL2*), which are critical for collagen processing. These findings align with previous proteomic studies of eggshell membranes. Differential expression analysis revealed 181 genes that differed significantly between the translucent and opaque groups, with 92 genes upregulated and 89 genes downregulated in the translucent group. Among these, ENSGALG00000053886, lipoprotein (*APOH*), calcium-binding protein (*CALB1*), glial fibrillary acidic protein (*GFAP*), proteolipid protein (*RNF144A*), and ENSGALG00000048109 were notably abundant.

Combining phenotypic traits such as microstructure and toughness, further screening identified *G0S2*, *CALB1*, *CACNA2D2*, *SLC4A1*, *SLC51A*, and *LAMP3* as significantly downregulated in the translucent group, while *OVoDA1*, *APOH*, *PLP1*, and *KIF26A* were significantly upregulated. Lipoprotein (*APOH*) is known to influence egg white formation (20), and calcium-binding protein (*CALB1*) may impact eggshell translucency by affecting eggshell quality (21, 22).

Two key genes, *PLP1* (proteolipid protein) and *APOH* (apolipoprotein), are involved in lipoprotein metabolism and lipid regulation (23, 24). Lipoproteins and liver-synthesized proteins are critical for egg production (25, 26). We hypothesize that disruptions in lipid synthesis pathways in the liver of translucent individuals may alter the eggshell membrane structure or protein composition, leading to membrane thinning and fiber structural differences, which contribute to translucency. *KIF26A*, a kinesin family protein, inhibits cell growth signaling pathways (27). *OVoDA1*, a cysteine-rich host defense peptide and member of the avian-specific defensin family, is abundant in egg whites and plays a central role in innate immunity, with broad antimicrobial activity against bacteria, fungi, and viruses (28). Additionally, *OVoDA1* is involved in immune regulation and barrier protection, such as activating immune cells, preventing infections, and maintaining intestinal homeostasis (29). Some studies suggest that defensins, including *OVoDA1*, can inhibit lipase activity (30). The higher expression of *OVoDA1* in both groups suggests its potential role in lipid metabolism, leading to differences in eggshell membrane chemical composition and physical structure during formation. This dysfunction in the isthmus of the translucent group may result in eggshell membrane differences and translucency.

The downregulation of *CACNA2D2*, involved in calcium binding and transport, suggests its role in calcium deposition during eggshell formation, potentially influencing translucency severity (31). *SLC4A1* and its family members are critical for transporting Na^+^, Ca^2+^, and Fe^3+^ ions across cell membranes (32, 33), and their dysregulation may impair calcium, sodium, and iron transport pathways, affecting eggshell membrane structure or protein composition (34). *G0S2*, a small basic protein, is involved in proliferation, apoptosis, inflammation, metabolism, and carcinogenesis (35, 36). Its abundance in metabolically active tissues like fat and liver suggests its role as a regulator of triglyceride catabolism (37), consistent with the observed upregulation of *OVoDA1*. These findings collectively suggest that disruptions in lipid metabolism and calcium deposition in the isthmus of the translucent group may lead to eggshell membrane thinning and translucency. However, further investigation is required to fully elucidate the molecular mechanisms underlying these observations.

Furthermore, while our RNA-seq analysis provides a comprehensive transcriptome profile, the absence of orthogonal validation (e.g., qPCR or proteomic analysis) for key differentially expressed genes represents a technical limitation. Although stringent bioinformatics pipelines were applied to ensure data quality, future studies incorporating experimental validation would further strengthen the biological relevance of these findings.

### Proteome

According to the protein quantification results, albumin (*ALB*), RNA-binding motif protein 3 (*RMB3*), and immunoglobulin-like polypeptide 1 (*IGLL1*) exhibited higher abundance in the opaque group, while lysyl oxidase-like 2 (*LOXL2*), ovalbumin A1 (*OVoDA1*), histone *H2A*, collagen (*COL1A2*), sulfhydryl oxidase (*QSOX1*), sulfhydryl reductase (*TXN*), peptidyl prolyl isomerase C (*PPIC*), and prolyl 4-hydroxylase subunit α2 (*P4HA2*) were relatively abundant in both groups. These findings align with transcriptomic results, which indicate that chicken isthmus tissues contribute to egg white glucose provision (38) and that functional proteins, such as *P4HA2*, may facilitate glucose metabolism and utilization (39). Sulfhydryl oxidase (*QSOX1*) and sulfhydryl reductase (*TXN*) are enzymes that regulate dimer cross-linking, suggesting that isthmus-derived proteins are involved in fibril formation within the eggshell membrane. The high expression of lysyl oxidase likely enhances the interaction between fibrillar proteins and collagen in the eggshell membrane, thereby influencing its structure.

In this study, 565 differential proteins were identified, with 249 upregulated and 316 downregulated. These proteins were primarily associated with catalytic activity, biological regulation, carbohydrate metabolism, amino acid metabolism, and lipid metabolism. The isthmus tissue is the primary site of eggshell membrane formation (40), and the eggshell membrane is composed of mucins, lipids, sugars, and trace minerals such as calcium and magnesium. The enriched pathways related to amino acid and lipid metabolism may reflect the requirement of these processes for eggshell membrane formation (41). Additionally, the isthmus tissue injects glucose into the egg white and secretes papillae onto the eggshell membrane, which play a critical role in eggshell calcification (42). This may explain the observed enrichment of proteins in molecular binding, biological process regulation, and protein complex pathways. Protein interaction network analysis revealed key roles for acyl-CoA dehydrogenase family member 8 (*ACAD8*) and acyl-CoA oxidase 1 (*ACOX1*), both of which are involved in lipid metabolism (43, 44). These findings suggest that lipid metabolism is closely associated with eggshell membrane formation and may influence the development of translucency.

In this study, comparative proteomic analysis of the translucent and opaque groups revealed significant differences in protein expression associated with eggshell membrane formation in the oviductal isthmus. Specifically, *SLC4A1*, *APOA1*, *APOA4*, *KRT7*, and *KRT18* were significantly upregulated in the translucent group. These proteins are involved in diverse biological processes, including transmembrane transport, lipid metabolism, and cytoskeletal organization. For instance, *SLC4A1*, a transporter involved in transmembrane ion transport, was enriched in GO terms related to secondary transmembrane transport protein activity. The upregulation of apolipoproteins such as *APOA1* and *APOA4* suggests potential impairments in lipid metabolism (45), which may contribute to structural variations in the eggshell membrane, ultimately leading to its thinning and the formation of translucency. This finding aligns with transcriptomic results, further supporting the hypothesis that disrupted lipid metabolism plays a role in eggshell membrane alterations.

*KRT7* and *KRT18*, both keratin proteins, were also significantly upregulated in the translucent group. *KRT18*, a cytoskeletal protein, is essential for maintaining cellular and tissue integrity and is involved in cell division and apoptosis (46). Previous studies have reported that overexpression of *KRT18* may lead to adverse effects, including tumor-like cellular behavior (47). The elevated expression of *KRT18* and *KRT7* in the translucent group may indicate accelerated apoptosis in certain cells, potentially disrupting eggshell membrane formation and contributing to translucency.

Conversely, *SPINK7*, *HSPA5*, *FBN1*, *OVST*, *AVBD11*, and *AVBD12* were significantly downregulated in the translucent group. *SPINK7*, a protein with carbohydrate-binding functions, is involved in the negative regulation of endopeptidase activity and steroid hormone processes (48). It has been suggested that *SPINK7* may play a role in the early stages of eggshell biomineralization (49), and its downregulation could impair this process, leading to eggshell membrane defects and translucency.

*HSPA5*, also known as glucose-regulated protein 78 (*GRP78*), is a molecular chaperone involved in lipid metabolism and cellular stress responses. Studies have shown that *GRP78* is essential for adipogenesis, lipogenesis, and postnatal growth in mice, with its inhibition significantly reducing lipid content (50). *HSPA5* is also critical for insulin secretion, as its downregulation leads to reduced insulin levels (51). The decreased expression of *HSPA5* in the translucent group may impair lipid metabolism and adipogenesis, potentially altering the composition and structure of the eggshell membrane. This could result in reduced lipoprotein levels and fewer fibrous structures within the membrane, contributing to its thinning and translucency.

*FBN1*, which encodes fibronectin 1, was also downregulated in the translucent group. Fibronectin 1 is essential for maintaining the elasticity of the eggshell membrane (52, 53). Its reduced expression may lead to a loss of elastic tissue structure, decreasing the membrane’s elasticity and toughness. Additionally, *FBN1* is associated with the production of asprosin, a glycogenic adipokine that regulates carbohydrate and lipid metabolism (54). The downregulation of *FBN1* in the translucent group suggests impaired metabolic regulation, further supporting the hypothesis that lipid metabolism disruptions contribute to eggshell membrane thinning and translucency.

*AVBD11* and *AVBD12*, members of the avian β-defensin family, were also downregulated in the translucent group. *AVBD11* is an atypical defensin expressed primarily in the oviduct but also found in the eggshell and eggshell membrane. It plays a critical role in embryonic growth regulation, antimicrobial immunity, and angiogenesis (55, 56). *AVBD11*’s presence in the eggshell membrane may contribute to its structural integrity and resistance to bacterial penetration. Its downregulation in the translucent group could impair these functions, leading to structural defects and translucency. *AVBD12*, another innate immune regulator, inhibits or kills pathogens (57, 58). The reduced expression of *AVBD11* and *AVBD12* in the translucent group may weaken the immune function of the eggshell membrane, resulting in incomplete or chemically altered structures that contribute to translucency.

Overall, the proteomic analysis revealed that differential proteins in the translucent group were primarily involved in immunity, lipid metabolism, and tissue structure. These findings are consistent with both the eggshell membrane proteome and isthmus transcriptome results, further supporting the hypothesis that disruptions in lipid metabolism and immune function contribute to eggshell membrane thinning and translucency.

### Conjoint analysis

A total of 16,666 genes were identified in the transcriptome of this experiment, representing 97.99% of the original chicken database. Proteomic analysis of the isthmus identified 5,882 proteins, while the eggshell membrane proteome identified 900 proteins. In the integration of isthmus proteome and transcriptome data, 5,882 transcriptomes were quantified to corresponding proteins, accounting for 35.29% of the identified genes. Similarly, in the integration of eggshell membrane proteome and transcriptome data, 900 transcriptomes were quantified to corresponding proteins, representing 5.40% of the identified genes. The experimental material for this study was the dwarf white hen, a pure breed of egg-laying hens with relatively low population variation, resulting in a smaller number of differential genes and proteins. The isthmus is the site of eggshell membrane production; however, the eggshell membranes analyzed in this study were collected from eggs laid by dwarf white hens, and differences in the timing of gene regulation and protein expression may contribute to discrepancies between the proteome and transcriptome of the isthmus.

The transcriptome and isthmus tissue proteome in this study showed a moderate correlation, consistent with previous studies (59, 60). This may be attributed to differences in the methods used for proteome and transcriptome identification. Earlier studies often employed 2D electrophoresis for proteome identification, which typically yielded a smaller number of high-abundance proteins. In contrast, this study utilized the advanced DIA (data-independent acquisition) technique, which provided high-accuracy identification of a larger sample of proteins. As a result, the correlation coefficients obtained in the transcriptome-proteome correlation tests were lower than those reported in studies using 2D electrophoresis (61). For instance, Pan et al. (62) observed a low degree of association between differential genes and differential proteins in red sweet orange, and Huang et al. similarly reported weak correlations between transcriptome and proteome data. These findings highlight the complexity of gene expression regulation.

In addition to methodological differences, the distinct half-lives of proteins and mRNAs, as well as protein degradation, can influence the correlation between transcriptome and proteome (63). These factors underscore the notion that mRNA transcription and protein expression are only partially correlated, and that mRNA levels do not fully reflect protein expression levels. This complexity indicates that transcriptome and proteome regulation involve distinct mechanisms, and that a comprehensive understanding of molecular regulation requires the integration of both datasets.

The results of the joint analysis revealed that not all mRNA and protein levels exhibited significant changes. This is primarily due to the intricate nature of transcriptional and post-transcriptional regulation (64, 65), which results in differences in protein and mRNA half-lives, synthesis rates, and quantities (66). Additionally, limitations inherent to the DIA-MS method, such as reduced quantification accuracy when analyzing complex MS/MS spectra generated by a wide isolation window, may also contribute to these discrepancies (22). The translatome, which bridges the transcriptome and proteome, is particularly suited for quantifying translated gene products and indirectly assessing protein expression at the genomic level (67). These findings emphasize the importance of integrating transcriptomic and proteomic data to gain a more comprehensive understanding of molecular regulatory mechanisms.

## Conclusion

The transcriptome results indicated that the causes of eggshell translucency might be associated with various biological processes, including metabolism, signaling pathways, immune system function, molecular binding, transport, and catabolism. Seven potential candidate genes, such as *CACNA2D2* and *KIF26A*, were identified. These genes may play roles in calcium transport and cell signaling, potentially influencing the structural integrity of the eggshell membrane.

Proteomic analysis revealed that the formation of translucent eggshells is linked to catalytic activity, biological regulation, carbohydrate regulation, amino acid metabolism, and lipid metabolism. Key proteins identified included *SLC4A1*, *APOA1*, and *APOA4*, which are involved in ion transport and lipid metabolism. These proteins may affect the composition and structural integrity of the eggshell membrane.

Integrated transcriptomic and proteomic analysis identified a total of 42 common differential proteins and genes, including *CA2*, *LUZP2*, *OVA*, *OVALY*, *OVST*, and *AVBD11*. These genes and proteins are likely involved in the formation and quality of the eggshell membrane.

The combined transcriptomic and proteomic analysis of isthmus tissue provides novel insights into the regulatory mechanisms governing eggshell membrane quality. The identified genes offer valuable molecular genetic references for the selection and breeding of hens with optimal eggshell membrane characteristics.

## Data Availability

The data presented in the study are deposited in the NCBI repository under the accession number GSE297167.

## Footnotes

^1^ http://www.r-project.org/
